# Measuring population health and quality of life: Developing and testing of the significant quality of life measure (SigQOLM)

**DOI:** 10.1016/j.heliyon.2023.e22668

**Published:** 2023-11-29

**Authors:** Mohamad Adam Bujang, Wei Hong Lai, Yoon Khee Hon, Eileen Pin Pin Yap, Xun Ting Tiong, Selvasingam Ratnasingam, Alex Ren Jye Kim, Masliyana Husin, Yvonne Yih Huan Jee, Nurul Fatma Diyana Ahmad, Jamaiyah Haniff

**Affiliations:** a Clinical Research Centre, Sarawak General Hospital, Ministry of Health Malaysia, Kuching, Sarawak, Malaysia; b Institute for Clinical Research, Ministry of Health Malaysia, Shah Alam, Selangor, Malaysia; c Psychiatric Department, Sarawak General Hospital, Ministry of Health Malaysia, Kuching, Sarawak, Malaysia; d Quality Unit, Sarawak General Hospital, Ministry of Health Malaysia, Kuching, Sarawak, Malaysia; e Radiotherapy and Oncology Unit, Sarawak General Hospital, Ministry of Health Malaysia, Kuching, Sarawak, Malaysia; f Heart Center, Sarawak General Hospital, Ministry of Health Malaysia, Kuching, Sarawak, Malaysia; g Malaysian Health & Performance Unit, Ministry of Health Malaysia, Putrajaya, Wilayah Persekutuan, Malaysia

**Keywords:** Health, Questionnaire development, Quality of life, Reliability, Validity

## Abstract

Quality of life (QOL) should ideally be determined by a broader spectrum of measurable parameters. This study aims to develop and validate a study instrument that is designed to determine a holistic measure of health and non-health aspects of QOL, and it is called the ‘Significant Quality of Life Measure’ (SigQOLM). This study involves five phases which aim to (i) explore and understand the subject matter content, (ii) develop a questionnaire, (iii) assess its content validity and face validity, (iv) conduct a pilot study, and lastly (v) perform a field-test by using the questionnaire. For the field-testing phase, a cross-sectional study was conducted which elicited responses from healthcare workers via a self-administered survey for all the SigQOLM items. Based on the results, the overall framework of the SigQOLM consists of four elements, 18 domains with 69 items. The element of “Health” is measured by nine domains, while “Relationships”, “Functional activities, and “Survival” are measured by three domains respectively. The SigQOLM has been developed successfully and then validated with a high level of reliability, validity, and overall model fit. Therefore, the SigQOLM will provide researchers and policymakers another viable option to elicit a more comprehensive outcome measure of QOL which shall then enable them to implement specific interventions for improving the QOL of all the people, both healthy or otherwise.

## 1 Introduction

Quality of life (QOL) is a complex multidisciplinary and multidimensional concept that concerns the overall state of ‘well-being’ for both the individual and society as a whole [1]. The World Health Organization (WHO) defines QOL as the assessment of an individual's perception of their position in life in the context of the culture and value systems in which they live and in relation to their goals, expectations, standards, and concerns [2]. QOL can also be perceived from a broader spectrum of interrelated considerations whereby it is regarded to be inherently a dynamic, multi-level, and complex concept, which arises from its reflection of objective, subjective, macro-societal, and micro-individual, positive, and negative influences that often interact together [3].

Based on the abovementioned definition of QOL, an assessment of QOL shall not be merely contingent upon giving due consideration to health factors alone. For example, patients with diabetes mellitus may need to change his/her diet regime in order to achieve optimal glycemic control and this will certainly affect their QOL [4]. Another common example we can routinely find is when a person's future expectations in life can possibly be swayed by an uncertain event such as the global impact of a serious pandemic like COVID-19 [5]. In addition, COVID-19 has affected not only people's health status but also the safety of their daily living in a social, psychological, and economical sense [[5], [6], [7], [8]]. It must be emphasized that most of the currently available QOL scales do not address these elements adequately.

Why do we need to measure QOL? It should be clear by now that the provision of medical treatments, the development of healthcare facilities and other related infrastructure, and the implementation of healthcare policies are being initiated by the government or sometimes non-government agencies, all of which are meant to serve all people in the community by improving or maintaining their optimal QOL [9,10]. While all these efforts for achieving optimal health status are continuously progressing, the effort to measure the people's QOL has also become necessary correspondingly. All researchers or policymakers shall then be better equipped to monitor the people's QOL and also to subsequently make an effort to improve their QOL. In particular, the researchers need the right tool to measure QOL which shall enable them to identify an ideal intervention or treatment program, so that it can be introduced and then widely implemented for improving the QOL of all people [11].

Most of the available QOL measures usually emphasize solely on an assessment of a patient's health-related QOL as these specific QOL measures are designed to measure a patient's health outcomes [[12], [13], [14], [15]]. Some of these study instruments are used for determining a general health and well-being status for its QOL measure [2,[16], [17], [18]]. On another note, there are also various other disease-specific instruments that are used for determining QOL measures for specific diseases [[19], [20], [21]]. A previous study attempted to measure QOL beyond the usual ‘health’ domain, and only two items out of a total of sixteen items were identified to represent the indicator for the measurement of the ‘health’ domain [22]. A vast majority of these scales mentioned above were developed more than 20 years ago [[12], [13], [14], [15], [16], [17], [18], [19], [20], [21], [22]].

It should be borne in mind that the various elements or domains of QOL can always be expanded further to suit the definition of ‘QOL’ which is intended to accommodate a broader spectrum of many other related variables for a variety of differing contexts. It is necessary to empirically explore the utility of these additional elements which can then be demonstrated to be both reliable and valid for representing an ideal measure for the universal scale of QOL worldwide. Moreover, most people will probably need a much-improved QOL scale with a broader coverage within the context of their societal and cultural perspectives. In view of this, it has now become imperative for people to collectively assess the viability of a more comprehensive view or perception of their QOL rather than merely be focusing on health considerations alone.

Therefore, the present study aims to develop a novel version of the quality-of-life scale which will be able to incorporate a broader spectrum of elements and domains which shall be named herein as the ‘Significant Quality of Life Measure’ (SigQOLM). More specifically, this study aims to develop and then test the psychometric properties of the SigQOLM (i.e. reliability and validity) for measuring health and non-health aspects of QOL among the general population (both healthy and otherwise).

### 1.1 Material and methods

#### 1.1.1 To develop the questionnaire

This study generally adopted the method from previous studies to develop a questionnaire that comprised five sequential phases [23,24]. More specifically, the process of development and testing of the SigQOLM involve; (i) to study the background subject matter, (ii) to develop the specific items of the questionnaire, overall structure, and format of the questionnaire, (iii) to assess its content and face validity, (iv) to conduct a pilot study, and (v) a field-test by using the SigQOLM items.

##### 1.1.1.1 To study the background subject matter

The authors took the first step by conducting an extensive and thorough literature review of all relevant studies pertaining to the concept of QOL. This is meant to upgrade the level of knowledge of the core subject matter content by exploring the theories and concepts of QOL. This process is necessary before develop the overall conceptual framework for the ‘SigQOLM’. After finalize the framework for SigQOLM, the authors then proceed to develop all the items which are subsequently categorized into 21 different domains. An initial proposal of the establishment of 21 domains of ‘SigQOLM’ is based upon its overall conceptual framework. They are now tabulated in Table 1 along with their respective justifications. The ‘SigQOLM’ aims to provide a new scale for measuring a generic QOL based on a list of various health and non-health matters. The health-related parameters in SigQOLM are measured by “Health” elements and the non-health parameters or trivially referred to as ‘personal well-being’ are measured by elements “Relationship”, “Functional activities”, and “Survival”.

**Table 1:** Proposed domains for ‘SigQOLM’ and the justifications.

No.	Elements	Domains	Definition	References (in relation to QOL)
1	HEALTH *(as the condition of being sound in body, mind, or spirit)*	*Physical pain*	A feeling of any unpleasant physical sensation in any part of the body caused by illness or injury	[2,17,18,25]
2		*Physical energy*	The strength and vitality required for a sustained physical activity	[2,17,18,26]
3		*Independent*	The ability of a person to act independently or be able to survive without any assistance from others	[17,18,27,28]
4		*Emotional symptoms*	The existence of an unpleasant emotion due to experiencing any unfavorable and/or unexpected conditions	[2,17,18,29]
5		*Sleep quality*	Quality of sleep of a person	[2,30,31]
6		*Eating regime*	Eating problems and/or eating disorders experienced by a person	[4,32,33]
7		*Mobility*	The ability of a person to move by himself/herself	[17,18]
8		*Body image*	The overall perception or feeling of satisfaction towards the stature or physical build of a person	[2,34,35]
9		*Perception of future health*	A strong belief something will happen (good or bad) related to his/her future health condition	[[36], [37], [38]]
10	RELATIONSHIPS *(a concept that portrays the way by which two or more people or things are being related or interrelated)*	*Family relationships*	The way in which a person has connected with his/her close family members	[2,39,40]
11		*Friendships*	The way in which two or more people (not family members) are connected	[2,17,18]
12		*Religiosity*	A person's ability to form a meaningful relationship with God and religion	[41,42]
13	FUNCTIONAL ACTIVITIES *(as a measure of the extent to which a person is able to perform a state of being*active *in behaviour or*actions*)*	*Self-care*	The ability of a person to perform self-care activities independently	[17,18]
14		*Social life*	The ability of a person to be engaged in usual indoor or outdoor activities with family members, friends or colleagues	[2,17,18]
15		*Usual activities*	Perception of a person's routine set of activities that is repeated regularly	[18,43]
16		*Perception of time usage*	Perception of a person regarding his/her time usage	[44,45]
17	SURVIVAL *(as the act or fact of living for the continuation of life)*	*Safety*	An unpleasant emotion caused by the surrounding and/or any other factors related to the environment	[46,47]
18		*Eating sufficiency*	Adequacy of daily eating needs	[[48], [49], [50]]
19		*Living environment*	Any unpleasant emotion caused by the living environment	[46,47]
20		*Fulfillment of basic needs*	The capability to obtain access to basic needs such as food, water, clothes, and shelter for living	[2,50]
21		*Perception of future conditions*	A strong belief that a future event will happen in a person's life	[[36], [37], [38]]

##### 1.1.1.2 Overall process of scale development for a questionnaire

Table 1 shows a list of all the possible domains of the construct of SigQOLM along with their respective definitions and supporting citing references. It is initially proposed to empirically hypothesize that all these domains are categorized into four main elements; namely “Health”, “Relationships”, “Functional activities” and “Survival”. By adopting this type of categorization, it is then possible to identify a total of nine domains for “Health”, five domains for “Survival”, four domains for “Functional activities” and three domains for “Relationships”. Since each domain has already been pre-specified to consist of five items during a preliminary round of item generation, this adds up to a total of 105 items initially.

Hence, the aim of this study instrument SigQOLM is to evaluate how each study respondent feels about his/her QOL which shall be measured by both the ‘health’ and ‘non-health’ aspects of his/her daily living during the past two weeks. Thus, the respondents shall be asked how frequently certain events might have happened in his/her life over the past two weeks. An ordinal scale that is based on a five-point Likert scale such as “Never”, “Seldom”, “Sometimes”, “Normally” and “Always'' shall be used to capture these responses.

##### 1.1.1.3 Assessment of content validity and face validity

A panel of five subject matter experts (SMEs) consisting of a psychiatrist (n = 1), an epidemiologist (n = 1), a senior medical officer (n = 1), and two senior researchers with Ph.D. qualifications (n = 2) had assessed its content validity. There are basically two important measures that are designed to determine its content validity, the first being the extent to which each item can be measurable for defining a trait and the second is the ability to represent all aspects of a trait by a defined set of items or indicators.

All the SigQOLM items were first developed in English. All the questions were translated into Malay by using forward translation and then the Malay version of this study instrument was also translated back to English by using backward translation. Hence, the study group conducted a careful evaluation of both the English and Malay versions of the questionnaire, and subsequently, its face validity was assessed.

During an assessment of its face validity, at least ten respondents chosen from among the healthcare workers were then elected to review and provide their feedback on the SigQOLM items. For the purpose of linguistic validation, a cognitive debriefing process was conducted before the determination of its face validity. Following this debriefing process, all the SigQOLM items are then ready to be administered to the respondents. Each of the respondents was personally interviewed about the scope of the study, the instructions for filling in their responses, the response format itself, and all the instrument items.

##### 1.1.1.4 Pilot study

After the conduct of both content validity and face validity were successful, then a pilot study was conducted among 30 respondents who consist of healthcare workers from various job positions and professional backgrounds. The SigQOLM has two versions, English and Malay and the respondents can choose the version that they wish to answer. The aim of the pilot study is to test the reliability of the SigQOLM items; and its domains. Test-retest reliability was then assessed with a time lag of two weeks between the two rounds of reliability testing. The goal towards attaining adequate reliability and consistency for a new instrument is to achieve a minimum value of 0.70 for the kappa statistic, a measurement of interrater reliability which shall determine the level of kappa for each item, and also a minimum value of 0.65 for the Cronbach's alpha, a measurement of its internal consistency among all its target domains. After all the results from the pilot study were excellent, then a field test was conducted to determine the validity of the SigQOLM.

##### 1.1.1.5 Field-testing of SigQOLM

The field-testing of SigQOLM was conducted based on a cross-sectional study which is also a self-administered survey. The respondents are free to choose either to answer based on the English or the Malay versions of the SigQOLM. The study sample was recruited from both Sarawak Heart Center and Sarawak General Hospital. Both hospitals are under the administration of the Ministry of Health, Malaysia. The inclusion criteria for recruiting the study respondents for this self-administered survey are: (i) all workers currently working in a healthcare setting including permanent, contract, and temporary staff, (ii) age of at least 18 years old and above, and (iii) agree to provide consent for participating in the study. Respondents who are unconscious, too sick, or have an unstable mental condition during the period of recruitment were excluded from this study because they have to fill in their responses by answering an online questionnaire.

#### 1.1.2 Data collection

A snowball sampling technique was used to obtain the study sample, which involved contacting prospective study respondents via Whatsapp or email. The recruitment process was based on voluntary basis. The researchers sent a Whatsapp message or an email to each department in the hospital and the head or manager of the department would then share and assign the message to their staff by providing a link to the google form of the ‘SigQOLM’ questionnaire for them to fill in. Data from respondents were collected within two months from February 2022 until the end of April 2022.

#### 1.1.3 Sample size planning

The sample size requirement for this study was based on a rule of thumb for Exploratory Factor Analysis (EFA). Initially, it is presumed that each latent variable or domain in the ‘SigQOLM’ can give rise to no more than 50 constituent items. This means that based on the rule of thumb of using the 5:1 ratio for its sample size determination, the minimum sample size of 50 x 5 = 250 respondents would then be required [51,52]. In order to make allowances for the effect of a non-response rate of at least 30.0 %, it is therefore necessary for this study to accommodate the possibility of non-response by inflating its sample size by targeting to recruit at least 250/0.7 or 358 respondents.

#### 1.1.4 Statistical analysis

The main aim of this study was to develop a new scale that can assess a holistic measure of QOL which is designed to take into account a wide variety of other relevant considerations. Descriptive statistics were applied to describe the socio-demographic profiles of all the study respondents. Cronbach's alpha and Kappa agreement tests were used to assess the internal consistency and the agreement of the domains. Finally, both exploratory factor analysis (EFA) and confirmatory factor analysis (CFA) were applied to determine the construct validity and also to evaluate the domains and overall model fitness of SigQOLM to the observed data. EFA was conducted by utilizing Maximum Likelihood Estimation (MLE) as the method for factor extraction along with Varimax for its factor rotation.

The orthogonal rotation (i.e. Varimax) method was chosen instead of the oblique rotation method due to two reasons. First, this study is based on an underlying assumption that the domains within the same element are not necessarily correlated. For example, a person with insomnia who experiences a sleep problem does not necessarily have any problems in terms of his/her functional independent, overall mobility, daily eating habits, and perception of his/her body image. In terms of relationships, those who have problems establishing an amicable relationship with their family members are not necessarily having any problems with their friends. For survival, those who have a sufficient supply of basic daily needs might not necessarily feel safe, especially during a pandemic period such as COVID-19. Although there is always a possibility for an association to exist between various domains within the same element, it shall be fair to presume there is no pre-existing correlation between the various domains of this SigQOLM instrument for the purpose of developing its factor structure.

Another reason for not selecting the oblique method for factor rotation in this study is to ensure model simplicity in the results of this study; as the oblique solution best furnishes what might be called ‘factor simplicity’, while the orthogonal solution provides what scholars might designate as ‘model simplicity’ [53]. In this study, the framework of SigQOLM was developed first before conducting EFA to determine its factor structure. The elements that were included in the initial framework of SigQOLM were deemed to be highly relevant for explaining the broader spectrum of QOL and each domain under their respective elements was also carefully selected based on existing published literature (Table 1). Hence, each of these identified domains shall serve an important purpose by representing an independent indicator for the measurement of a person's current health condition. Due to this reason, an orthogonal rotation is preferable to oblique rotation and hence it shall be selected for use as a factor rotation method in this study.

The factor solution was derived by basing on the retention of any factors with an eigenvalue of >1 (based on the Kaiser criterion). During this process, it is necessary to delete any items which cross-load too highly between the factors (i.e. the maximum cross-loading allowed shall be within a limit of ±0.20) or any items with too low a factor loading of less than 0.40. All problematic items shall be deleted one by one until the ideal construct has finally emerged.

After using EFA to identify the factor structure which is inherently present in a set of variables, the overall model fit was assessed by using CFA where various indicators such as the Chi-square test (<3.0), Root Mean Square Error Approximation (RMSEA <0.08) and Standardized Root Mean Square Residual (SRMR <0.08) were estimated [54]. All analyses for model fit statistics were carried out using SPSS (IBM Corp. Released 2011. IBM SPSS Statistics for Windows, Version 20.0. Armonk, NY: IBM Corp.) and AMOS (Arbuckle, J. L. (2006). Amos (Version 7.0) [Computer Program]. Chicago: SPSS.)

#### 1.1.5 Ethical and regulatory considerations/informed consent

The recruitment of study respondents was based purely on voluntary participation. Prior informed consent was obtained from all the study respondents before they were invited to fill in the online questionnaires. Only those study respondents who had given their consent to participate in the study would be allowed to answer the online questionnaire. Prior to data collection, informed consent was obtained from the respondents online. This study was approved by Medical Research and Ethics Committee (MREC) and the publication of the study results was also approved by the Director-General of the Ministry of Health, Malaysia. The ethical approval number for this study is NMRR ID-21-01979-XDL (IIR).

### 1.2 Results

#### 1.2.1 Respondents

There are 406 study respondents participated in the survey. The majority of these respondents ranging from 18 to 35 years (53.0 %), are Malay (34.0 %), are college graduates who are either a certificate or a diploma-holder (65.3 %), are married (68.0 %), and are of the nursing profession (63.3 %) (Table 2).

**Table 2:** Profile demographic of respondents.

Profile	Profile category	n	%
Gender			
	Male	74	18.2
	Female	332	81.8
Age group			
	18–35 years	215	53.0
	36–40 years	88	21.7
	41–50 years	89	21.9
	51–60 years	14	3.5
Ethnic			
	Malay	138	34.0
	Chinese	73	18.0
	Iban	79	19.5
	Bidayuh	83	20.4
	Melanau	12	3.0
	Others	21	5.1
Highest education level			
	Secondary school	19	4.7
	Graduated with certificate or diploma	265	65.3
	Graduated with undergraduate degree	91	22.4
	Graduated with postgraduate degree	22	5.4
	Graduated with specialty and/or fellowship training	9	2.2
Marital status			
	Married	276	68.0
	Single and never married	114	28.1
	Spouse passed-away	5	1.2
	Divorcee	11	2.7
Occupation			
	Specialist	21	5.2
	Medical Doctor	50	12.3
	Pharmacist	16	3.9
	Nurses	257	63.3
	Assistant Medical Doctor	11	2.7
	Other allied health officer	33	8.1
	Administrative Officer	8	2.0
	Others	10	2.5

#### 1.2.2 Validity of initial format for the SigQOLM construct that contains 105 items

To ensure that ‘SigQOLM’ has an adequate level of construct validity, it would be necessary to conduct EFA separately for each domain. The element of “Health” had maintained its nine domains with a reduced number of items from 45 to 33 items. The element of “Relationships” also maintained its three domains with a reduced number of items from 15 to 12 items. The element of “Functional activities” was constructed with only three domains due to the subsequent exclusion of the domain of “Usual activities”, resulting in a reduction of the total number of items from 20 to 13 items. Last but not least, the element of “survival” was constructed with only three domains by excluding the domains for both the elements of “Eating sufficiency” and “Living environment”, which reduced the total number of items from 25 to 11 items (Table 3).

**Table 3:** Exploratory factor analysis and confirmatory factor analysis of ‘SigQOLM’.

	Number of domains constructed based on Eigenvalue more than one	Factor loadings	Chi-square test <3.0	RMSEA <0.08	SRMR <0.08
2nd order model					
SigQOLM			2.130	0.053	0.070
1st order model					
Health	9	0.425–0.939	2.201	0.054	0.075
Survival	3	0.420–0.884	2.091	0.052	0.067
Relationships	3	0.638–0.864	2.101	0.052	0.068
Functional Activities	3	0.611–0.889	2.221	0.055	0.077

All the domains or items were excluded due either to a high cross-loading (i.e. more than a limit of ±0.20) or to a low factor loading (i.e. less than a minimum of 0.4). The elimination of the problematic domains and items was also meant to simplify the overall SigQOLM framework. For example, for the element of “Functional activities”, one of its domains, namely “Usual activities”, was deleted from the overall framework of SigQOLM since most of the items included in this domain had already fallen under various other domains. One of the main reasons for the removal of the domain of “usual activities” from the overall SigQOLM framework is that the items for the domain of “usual activities” are often too nonspecific with very broad coverage and as a result, the study respondents may often mistakenly perceive these items as being reflective of the other domains as well.

Likewise, for the element of “Survival”, two of its domains: namely “Eating sufficiency” and “Living environment” were also deleted because either their factor loadings were unacceptably too low, or they are actually cross-loading items with too high a loading on more than one component with only a very small difference of less than 0.10 between these loadings. In addition, the domain of “Fulfillment of basic needs” was already representing these two above-mentioned domains because the definition of “basic needs” shall already encompass the adequate provision of supplies of food, water, clothes, and a living place [49].

#### 1.2.3 Hypothesized framework

**Fig. 1:**
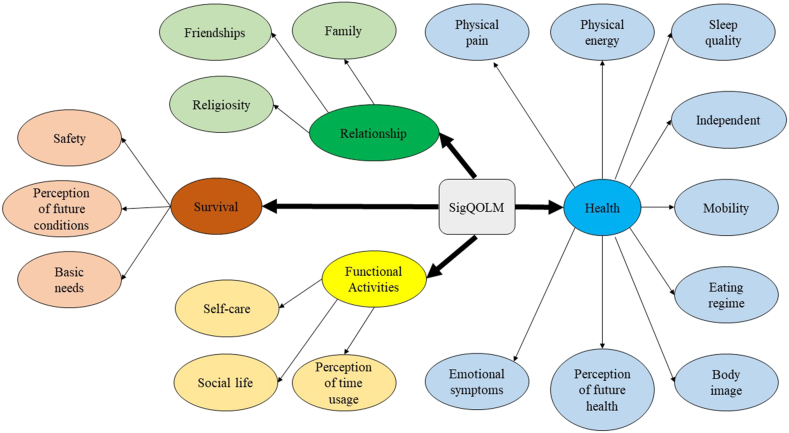
The framework of SigQOLM.

Based on the proposed factor solution, this study hypothesized the overall framework of SigQOLM as depicted in Fig. 1. From this framework, it is clear that the measurement of SigQOLM was based on four major elements with eighteen domains which means that there are 69 items altogether. The construct for the element of “Health” was determined to consist of a total of nine domains with a minimum factor loading of 0.425. Moreover, each of the three elements of “Survival”, “Relationships” and “Functional Activities” was constructed to consist of three domains respectively with a minimum factor loading of at least 0.420, 0.638, and 0.611 respectively. The overall model fit of the SigQOLM framework along with all the four major domains was found to be excellent based on the assessment of three model fit indicators such as Chi-square test <0.30, RMSEA <0.08, and SRMR <0.08. (Table 3).

#### 1.2.4 Reliability

The final version of SigQOLM which consisted of four elements and eighteen domains was reported to have excellent internal consistency reliability with a minimum value of 0.731 for its Cronbach's alpha (Table 4). However, one of the items falling under the element of “Health” reported a minimum value of 0.144 for its corrected item-total correlation, which is less than the recommended value of 0.2. Nevertheless, the authors decided to retain the item “I need equipment (e.g.: walking stick, wheelchair, etc.) to help me mobilize'' because the item highlighted an important indicator for the domain of “Mobility” and it was also an important indicator for the assessment of general health condition as a whole.

**Table 4:** Internal consistency of ‘SigQOLM’ main domains and sub-domains.

No.	Major domains/Sub-domains	no. of items	Cronbach's alpha	Min. CITC
	All	69	0.961	0.209
	*Health*	*33*	*0.934*	*0.144*
1	Physical pain	5	0.869	0.660
2	Physical energy	4	0.890	0.683
3	Emotional symptoms	3	0.866	0.722
4	Independent	3	0.806	0.623
5	Mobility	4	0.779	0.457
6	Sleep quality	4	0.876	0.666
7	Eating regime	2	0.749	0.599
8	Body image	4	0.914	0.736
9	Perception of future health	4	0.914	0.713
	*Functional Activities*	*13*	*0.875*	*0.307*
10	Social life	3	0.856	0.673
11	Self-care	5	0.865	0.565
12	Perception of time usage	5	0.913	0.693
	*Relationships*	*12*	*0.870*	*0.457*
13	Family relationships	4	0.821	0.620
14	Friendships	4	0.904	0.741
15	Religiosity	4	0.864	0.646
	*Survival*	*11*	*0.870*	*0.326*
16	Safety	4	0.845	0.509
17	Basic needs	3	0.731	0.442
18	Perception of future conditions	4	0.882	0.664

Note: Min. CITC refers to the minimum corrected item to total correlation.

### 1.3 Discussion

This newly-developed SigQOLM is specifically designed to elicit a more holistic measure of QOL and it aims to suit the needs (for eliciting a measure of QOL) of people from all walks of life. In pursuit of this goal, all the domains and items of SigQOLM are pre-specified to generate all the common measures of QOL from all people, regardless of their socio-demographic profiles. This empirical determination of all the domains, constructs, and items of SigQOLM shall aim to develop it as a generic study instrument that can universally cater to a valid assessment of ‘significant quality of life measure’ for a general study population of human subjects as broadly as possible.

#### 1.3.1 The overall framework of SigQOLM and its related domains

This study has successfully demonstrated that SigQOLM is a newly-developed instrument that is designed to elicit a holistic measure of QOL, and it is also found to be a reliable, and valid questionnaire. The final factor solution of SigQOLM has identified a total of 69 items with 18 domains which are further categorized into four elements. This study has introduced a new approach to measuring QOL by attempting to measure QOL beyond the usual ‘health element’ alone. Hence, it is indeed a novel approach to the measurement of QOL by incorporating other aspects of QOL in addition to its conventional health and/or health-related considerations.

Therefore, the measure of SigQOLM has been derived by the development of a new study instrument with a theoretical construct described by four elements which consist of “Health”, “Relationships”, “Functional activities” and “Survival”. In other words, a person with an excellent overall QOL is one who has an optimal health condition, is in a healthy relationship, is capable of being involved in functional activities, and also possesses the ability to survive or any other daily necessities that most people require for basic survival.

The first element is “Health”. This study has defined “Health” as the condition of being sound in body, mind, or spirit [55]. Meanwhile, the WHO defined health as “a state of complete physical, mental, and social well-being and not merely the absence of disease or infirmity” [56]. Therefore, to improve on the overall representation of the general “health” function, this study therefore, aims to measure the health status of a person by basing on nine domains that consist of (a) Pain, (b) Physical energy, (c) Emotional symptoms, (d) Independent, (e) Mobility, (f) Sleep quality, (g) Body image, (h) Diet regime, and (i) Perception of future health. It can therefore be regarded as an almost comprehensive measure of health outcomes as compared to all the previous measures of QOL [2,17,18]. For example, MOS SF36 did not include sleep quality and body image as the domains for a QOL measurement. Although WHOQOL-BREF has addressed both the domains of sleep quality and body image in two different items of this questionnaire, however, it manages to include only one item for each of the two domains while there are various other ways to portray to what extent a person feels about his/her satisfaction with sleep quality or body image.

The second element is “Relationships”. “Relationships” is a concept that portrays the way by which two or more people or things are related or interrelated [57]. In this instrument of SigQOLM, the element of “Relationships” is being measured based on (a) Family relationships, (b) Friendships, and (c) Religiosity or spiritual relationship with God. As human beings, all people will have the desire to communicate, interact, and live together with other people harmoniously. Hence, the extent to which a human being is able to nurture a harmonious relationship with other people shall have a bearing on his/her QOL [39,40]. Apart from the basic necessity of fostering a healthy relationship with people, an individual's religiosity or spiritual relationship with God is also important. A previous questionnaire, WHOQOL-Combi also addressed the religiosity or spiritual domain in QOL [58].

As humans, all of us are given a precious asset which is time. How we spend our time reflects who we are, who we will become, and whether or not our life is meaningful. This study has defined “Functional activities'' as a measure of the extent to which a person is able to perform a state of being active in behavior or actions [59]. The “Functional activities” measure activities such as (a) Self-care, (b) Social life, and (c) Perception of time usage. Self-care activities are the basic activities of all human beings for the purpose of taking care of oneself. Besides that, social activities are also considered to be under the same element where it measures how well a person can interact with others and participate in any social activities for our daily living. A major advantage of this newly-developed SigQOLM is that it has specifically allocated a wide variety of measures for the assessment of both ‘self-care’ and ‘social life’.

The overall scope of functional activities can be broadly classified as studying, working, leisure activities, and others. Since the SigQOLM is intended to be used by a vast majority of people, therefore all its domains and items should be adequately simplified and generalized so that all the domains and items can suit any individual. For example, working may often be regarded as part of an individual's functional activities. However, it is not true to say that everybody is working. For example, a retired person may no longer be working, so the term “working” is not relevant to him/her. In addition, it can be rather difficult to develop a construct that is able to capture many daily living activities and it is an even more complicated task to generalize this construct according to the specific contexts for different countries and different ethnicities or cultural backgrounds. Nevertheless, the most important requirement for the measurement of ‘SigQOLM’ is for all its domains to be contributing adequately towards an accurate and valid QOL measurement.

Therefore, the SigQOLM has now been developed to incorporate a specific domain for describing a person's perception of time usage in his/her life. This domain aims to measure to what extent a person makes use of his/her time wisely to be involved in any useful activities. What sort of activities we commonly engage in shall determine the quality of our current living conditions and therefore, those who use his/her time wisely for any useful activities shall be more likely to have a higher QOL as compared to those who are not engaging in any such activities. For example, those who are working very hard such as an entrepreneur will sooner or later be able to reap great rewards from his/her hard work.

Apart from good health, all human beings will also need other basic commodities to survive. Therefore, the final element is “Survival” and it consists of (a) Safety, (b) Fulfillment of basic needs, and (c) Perception of future conditions. The SigQOLM measure has defined the element of “survival” as the act or fact of living for the continuation of life [60]. Some people may equate the concept of “QOL in general” to financial stability and therefore a high QOL will be equivalent to having sufficient financial means in life which should be adequate to satisfy their material needs.

However, not all people can afford to be wealthy and be able to obtain whatever they want in life by using money as collateral. Therefore, the measurement of ‘SigQOLM’ has been developed to include a specific domain such as “fulfillment of basic needs” so that everybody will at least be able to address his/her basic requirements for survival in life. In other words, it is also a measure of being in possession of adequate resources for daily living which is based on the established measurement of a general standard, which can also be broadly regarded as an indicator of financial stability.

Having a safety net in life is also a requirement for QOL. Feeling safe can be regarded as a broadly overarching definition for the measurement of QOL such as feeling safe from contracting severe infectious diseases such as the pandemic COVID-19, feeling protected from the effects of crimes or wars from the surrounding areas, or even from natural disasters such as earthquakes or tsunamis. Safety is an important element because it could result in a life-or-death situation or in a potentially risky or dangerous situation at the minimum. Therefore, there is no doubt that the consideration of the safety aspect of life shall constitute an integral part of the QOL measure. The third domain for survival is an individual's own perception of his/her future condition. This domain is designed to measure to what extent a person is able to perceive his/her future situation or condition to be getting better or worse. Those who are currently having a better QOL shall have a fairly good perception of his/her future conditions [[36], [37], [38]].

#### 1.3.2 Scoring mechanism

SigQOLM assumes all items are equally important and have a similar weightage, and therefore, a simple scoring mechanism can be applied to score the overall framework of SigQOLM, as well as all its elements and domains. Each item is designed in such a way that a higher frequency of experiencing a particular situation or condition by an individual shall reflect a better QOL for him/her. Each item shall be measured based on a five-point Likert scale and thus the score will be allocated according to the following scale: Never = 4, Seldom = 3, Sometimes = 2, Normally = 1, and Always = 0. Then summation can be done by calculating the score for an overall assessment of the SigQOLM, along with its four elements and 18 domains. Therefore, the higher score of SigQOLM will directly indicate a better QOL of a person. The simple calculation of the scoring mechanism is presented in Table 5.

**Table 5:** Scoring mechanism for SigQOLM.

	Elements	Domains	Items	Scoring	Standardized score (%)
	*Health (n=33)*				
1		Physical pain (n = 5)	1,2,3,4,5	0 to 20	(Raw score/20) x 100
2		Physical energy (n = 4)	6,7,8,9	0 to 16	(Raw score/16) x 100
3		Emotional symptoms (n = 3)	10,11,12	0 to 12	(Raw score/12) x 100
4		Independent (n = 3)	13,14,15	0 to 12	(Raw score/12) x 100
5		Mobility (n = 4)	16,17,18,19	0 to 16	(Raw score/16) x 100
6		Sleep quality (n = 4)	20,21,22,23	0 to 16	(Raw score/16) x 100
7		Eating regime (n = 2)	24,25	0 to 8	(Raw score/8) x 100
8		Body image (n = 4)	26,27,28,29	0 to 16	(Raw score/16) x 100
9		Perception of future health (n = 4)	30,31,32,33	0 to 16	(Raw score/16) x 100
	Total Health score			0 to 132	(Raw score/132) x 100
	*Relationships (n=12)*				
10		Family relationships (n = 4)	1,2,3,4	0 to 16	(Raw score/16) x 100
11		Friendships (n = 4)	5,6,7,8	0 to 16	(Raw score/16) x 100
12		Religiosity (n = 4)	9,10,11,12	0 to 16	(Raw score/16) x 100
	Total Relationships score			0 to 48	(Raw score/48) x 100
	*Functional activities (n=13)*				
13		Self-care (n = 5)	1,2,3,4,5	0 to 20	(Raw score/20) x 100
14		Social life (n = 3)	6,7,8	0 to 12	(Raw score/12) x 100
15		Perception of time usage (n = 5)	9,10,11,12,13	0 to 20	(Raw score/20) x 100
	Total Functional activities score			0 to 52	(Raw score/52) x 100
	*Survival (n=11)*				
16		Fulfillment of basic needs (n = 3)	1,2,3	0 to 12	(Raw score/12) x 100
17		Safety (n = 4)	4,5,6,7	0 to 16	(Raw score/16) x 100
18		Perception of future conditions (n = 4)	8,9,10,11	0 to 16	(Raw score/16) x 100
	Total Survival score			0 to 44	(Raw score/44) x 100
					
	Total score for SigQOLM		1–69	0 to 276	(Raw score/276) x 100

#### 1.3.3 Recommendations and future studies

The SigQOLM was developed to measure QOL and the well-being of the people. In other words, the SigQOLM is suitable for use by both healthy and non-healthy people since the domains cover various spectrums based on health and non-health elements. Therefore, the SigQOLM can be used to measure the QOL of people from among all the various categories of human population so that researchers, policymakers (i.e. government agencies), and non-government agencies will be able to monitor and make changes to improve people's QOL and their well-being. Following that, validation studies of SigQOLM in various languages shall become necessary so that future studies may utilize SigQOLM to measure QOL and the well-being of people across different ethnicities, regions, and countries.

#### 1.3.4 Limitations of the study

The SigQOLM did not include a domain for assessing sexual life although it may be regarded as an important factor for QOL. It must be kept in mind that not all people are married; especially amongst adolescents, the widowed, and/or the divorced, who may actually not have a sex life at the outset. Hence, it would be meaningless for them to evaluate whether they are having a healthy sex life or not. Therefore, this study did not incorporate any domains or items that are related to a healthy sex life into the SigQOLM in order to avoid inadvertently introducing ‘missing values’ in the analysis. Hence, as far as a healthy sex life is concerned, it is necessary to adopt elsewhere or develop a totally different set of questionnaires for this purpose [61]. Likewise, the SigQOLM did not incorporate such domains as work relationships or job satisfaction because not all adults are actively working. However, the SigQOLM has included a new domain on “Perception of time usage” that measures people's perception of how they use and benefit their time.

Another limitation of this study is that its study sample only includes healthcare workers. An ideal sample to be obtained from the study population is a representative sample of the general population presenting with a wide variety of differing health conditions and socio-demographic profiles. Since the general population is too large and scattered, it will be too difficult to conduct the survey in the ideal setting, especially during the COVID-19 pandemic. Therefore, the study sample for this study was obtained from among the healthcare workers with an assumption that the sample which consists of people with various health conditions and socio-demographic profiles shall be deemed almost likely equivalent to the target population which is, in fact, the general population. The favorable results obtained from this study have further indicated that the construct of the domains has a strong scientific basis, the overall model fit has been found to be satisfactory, and the established theoretical framework for the SigQOLM instrument is also well substantiated by the existing supporting literature.

#### 1.3.5 Conclusions

The newly developed ‘Significant Quality-of-Life Measure’ has been successfully validated with an excellent level of reliability and validity as well as a satisfactory goodness-of-fit. This ‘SigQOLM’ consists of four elements, 18 domains, and 69 items. It can either be used for eliciting a holistic measure of QOL within a general human population by administering all four elements to the respondents or alternatively, it can also be tailored by a researcher for the assessment of QOL in a specific type of study population or for a specific research purpose via an administration of a few of its selected elements to the respondents. All the domains were further validated by both exploratory factor analysis and confirmatory factor analysis. Therefore, it is now recommended that researchers and policymakers use SigQOLM as an instrument to measure QOL and the well-being of all people, both healthy and otherwise. Future studies for the purpose of validating SigQOLM in various other languages among different populations may need to be conducted to test the suitability of SigQOLM for use in various types of study participants from among differing local populations and countries.

## Ethics statement

The written consent was obtained from all respondents. This study was approved by Medical Research and Ethics Committee (MREC) and the publication of the study results was also approved by the Director-General of the Ministry of Health, Malaysia. The ethical approval number for this study is NMRR ID-21-01979-XDL (IIR).

## CRediT authorship contribution statement

**Mohamad Adam Bujang:** Writing - review & editing, Writing - original draft, Validation, Supervision, Project administration, Methodology, Investigation, Formal analysis, Conceptualization. **Wei Hong Lai:** Writing - review & editing, Writing - original draft, Methodology, Investigation. **Yoon Khee Hon:** Writing - review & editing, Writing - original draft, Validation, Project administration, Investigation. **Eileen Pin Pin Yap:** Writing - review & editing, Writing - original draft, Validation, Software, Resources, Project administration. **Tiong Xun Ting:** Writing - review & editing, Writing - original draft, Methodology, Investigation. **Selvasingam Ratnasingam:** Writing - review & editing, Writing - original draft, Validation, Supervision, Investigation. **Alex Ren Jye Kim:** Writing - review & editing, Writing - original draft, Resources, Investigation, Data curation. **Masliyana Husin:** Writing - review & editing, Writing - original draft, Validation, Software, Formal analysis. **Yvonne Yih Huan Jee:** Writing - review & editing, Writing - original draft, Investigation, Data curation. **Nurul Fatma Diyana Ahmad:** Writing - review & editing, Writing - original draft, Investigation, Data curation. **Jamaiyah Haniff:** Writing - review & editing, Writing - original draft, Supervision, Conceptualization.

## Declaration of competing interest

The authors declare that they have no known competing financial interests or personal relationships that could have appeared to influence the work reported in this paper.

## References

[1] Petrovič F., Maturkanič P. (2022). Urban-rural dichotomy of quality of life. Sustainability.

[2] The WHOQOL Group (1998). Development of the world health organization WHOQOL-BREF quality of life assessment. Psychol. Med..

[3] Brown J., Bowling A., Flynn T. (2004). Models of quality of life: a taxonomy, overview and systematic review of the literature.

[4] Cerrelli F., Manini R., Forlani G., Baraldi L., Melchionda N., Marchesini G. (2005). Eating behavior affects quality of life in type 2 diabetes mellitus. Eat. Weight Disord..

[5] Ahorsu D.K., Lin C.Y., Imani V., Saffari M., Griffiths M.D., Pakpour A.H. (2020). Fear of COVID-19 scale: development and initial validation. Int. J. Ment. Health Addict.

[6] Singh J., Singh J. (2020). COVID-19 and its impact on society. Electron. res. j. soc. sci. humanities.

[7] Al-Shannaq Y., Mohammad A.A., Aldalaykeh M. (2021). Depression, coping skills, and quality of life among Jordanian adults during the initial outbreak of COVID-19 pandemic: cross sectional study. Heliyon.

[8] Al-Shannaq Y., Mohammad A.A. (2021). Psychological impacts during the COVID-19 outbreak among adult population in Jordan: a cross-sectional study. Heliyon.

[9] De Guimaraes J.C.F., Severo E.A., Junior L.A.F., Da Costa W.P.L.B., Salmoria F.T. (2020). Governance and quality of life in smart cities: towards sustainable development goals. J. Clean. Prod..

[10] Mo P.K.H., Wong E.L.Y., Yeung N.C.Y., Wong S.Y.S., Chung R.Y., Tong A.C.Y. (2022). Differential associations among social support, health promoting behaviors, health-related quality of life and subjective well-being in older and younger persons: a structural equation modelling approach. Health Qual. Life Outcomes.

[11] Bujang M.A., Lai W.H., Ratnasingam S., Tiong X.T., Hon Y.K., Yap E.P.P. (2023). Development of a quality-of-life instrument to measure current health outcomes: health-related quality of life with six domains (HRQ-6D). J. Clin. Med..

[12] Evans R.W., Manninen D.L., Garrison L.P., Hart L.G., Blagg C.R., Gutman R.A. (1985). The quality of life of patients with end-stage renal disease. NEJM. 1985.

[13] Croog S., Levine S., Tecta M., Brown B., Bulpitt C., Jenkins C. (1986). The effects of antihypertensive therapy on the quality of life. NEJM.

[14] MacKenzie C.R., Charlson M.E., DiGioia D., Kelley K. (1986). A patient-specific measure of change in maximal function. Arch. Intern. Med..

[15] Stead M., Brown J., Velikova G., Kaasa S., Wisløff F., Child J. (1999). Development of an EORTC questionnaire module to be used in health‐related quality‐of‐life assessment for patients with multiple myeloma. Br. J. Haematol..

[16] Hunt S.M., McEwan J., McKenna S.P. (1986).

[17] Ware J.E., Sherbourne C.D. (1992). The MOS 36-item short-form health survey (SF36). Conceptual framework and item selection. Med. Care.

[18] EuroQOL Group (1996).

[19] Lazarus R.S., Folkman S. (1984).

[20] Juniper E.F., O'Byrne P.M., Guyatt G.H., Ferrie P.J., King D.R. (1999). Development and validation of a questionnaire to measure asthma control. Eur. Respir. J..

[21] Bujang M.A., Adnan T.H., Mohd-Hatta N.K.B., Ismail M., Lim C.J. (2018). A revised version of diabetes quality of life instrument maintaining domains for satisfaction, impact, and worry. J. Diabetes Res..

[22] Flanagan J.C. (1982). Measurement of the quality of life: current state of the art. Arch. Phys. Med. Rehabil..

[23] Ahmad N.F.D., Ren Jye A.K., Zulkifli Z., Bujang M.A. (2020). The development and validation of job satisfaction questionnaire for health workforce. Malays. J. Med. Sci..

[24] Bujang M.A., Tan-Hui S. (2022).

[25] Skevington S.M. (1998). Investigating the relationship between pain and discomfort and quality of life, using the WHOQOL. Pain.

[26] Gill D.L., Hammond C.C., Reifsteck E.J., Jehu C.M., Williams R.A., Adams M.M. (2013). Physical activity and quality of life. J. Prev. Med. Public Health. 2013.

[27] Araujo I.L., Castro M.C., Daltro C., Matos M.A. (2016). Quality of life and functional independence in patients with osteoarthritis of the knee. Knee Surg. Relat. Res.

[28] Bozkurt U., Yılmaz M. (2016). The determination of functional independence and quality of life of older adults in a nursing home. Int. J. Caring Sci..

[29] Bujang M.A., Musa R., Liu W.J., Chew T.F., Lim C.T.S., Morad Z. (2015). Depression, anxiety and stress among patients with dialysis and the association with quality of life. Asian J Psychiatr. 2015.

[30] Stojanov J., Malobabic M., Stanojevic G., Stevic M., Milosevic V., Stojanov A. (2020). Quality of sleep and health-related quality of life among health care professionals treating patients with coronavirus disease-19. Int. J. Soc. Psychiatry..

[31] Zeitlhofer J., Schmeiser-Rieder A., Tribl G., Rosenberger A., Bolitschek J., Kapfhammer G. (2000). Sleep and quality of life in the Austrian population. Acta Neurol. Scand..

[32] Abraham S.F., Brown T., Boyd C., Luscombe G., Russell J. (2006). Quality of life: eating disorders. Aust. N.Z. J. Psychiatry..

[33] Jenkins P.E., Rienecke-Hoste R., Meyer C., Blissett J.M. (2011). Eating disorders and quality of life: a review of the literature. Clin. Psychol. Rev..

[34] Cox T.L., Zunker C., Wingo B., Thomas D.M., Ard J.D. (2010). Body image and quality of life in a group of African American women. Soc. Indic. Res..

[35] Nayir T., Uskun E., Yürekli M.V., Devran H., Çelik A., Okyay R.A. (2016). Does body image affect quality of life?: a population based study. PLoS One.

[36] Carr A.J., Gibson B.A., Robinson P.G. (2001). Is quality of life determined by expectations or experience?. BMJ.

[37] McKee K.J., Kostela J., Dahlberg L. (2015). Five years from now: correlates of older people's expectation of future quality of life. Res. Aging.

[38] Rosinha A.J., Silva B., Souza M., Junior R.C., Junior H.A. (2018). The influence of the future expectation on quality of life of cadets of Portuguese army. Millenium.

[39] Bennich B.B., Munch L., Egerod I., Konradsen H., Ladelund S., Knop F.K. (2019). Patient assessment of family function, glycemic control and quality of life in adult patients with type 2 diabetes and incipient complications. Can. J. Diabetes.

[40] Modanloo S., Rohani C., Azam S.F., Vasli P., Pourhosseingholi A. (2019). General family function as a predictor of quality of life in parents of children with cancer. J. Pediatr. Nurs..

[41] Rule S. (2007). Religiosity and quality of life in South Africa. Soc. Indic. Res..

[42] Moon Y.S., Kim D.H. (2013). Association between religiosity/spirituality and quality of life or depression among living alone elderly in a south Korean city. Asia Pac Psychiatry.

[43] Li C., Ford E.S., Mokdad A., Jiles R., Giles W.H. (2007). Clustering of multiple healthy lifestyle habits and health-related quality of life among U.S. adults with diabetes. Diabetes Care.

[44] Kabre F., Brown J.U. (2011). The influence of Facebook usage on the academic performance and the quality of life of college students. J. Media Commun. Stud..

[45] Wang W.C., Kao C.H., Huan T.C., Wu C.C. (2011). Free time management contributes to better quality of life: a study of undergraduate students in taiwan. J. Happiness Stud..

[46] Jakobsson U., Loneliness Hallberg I.R. (2005). fear, and quality of life among elderly in Sweden: a gender perspective. Aging clin. exp. res..

[47] Albouy D., Graf W., Kellogg R., Wolff H. (2014). Climate amenities, climate change, and american quality of life. J. Assoc. Environ. Resour. Econ..

[48] Watson D., Maître B., Whelan C.T., Russell H. (2017). Poverty, economic stress and quality of life – lessons from the Irish case. Int. Rev. Econ..

[49] Thongprasert N. (2018). Relationship between achievement of household accounting based on philosophy of sufficiency economy and quality of life. Int. J. Humanit. Soc. Sci..

[50] Gou Z., Xie X., Lu Y., Khoshbakht M. (2018). Quality of life (QOL) survey in Hong Kong: understanding the importance of housing environment and needs of residents from different housing sectors. Int. J. Environ. Res. Public Health.

[51] Bujang M.A., Ab-Ghani P., Soelar S.A., Zulkifli N.A. (2012). Sample size guideline for exploratory factor analysis when using small sample: taking into considerations of different measurement scales. Statistics in Science, Business, and Engineering (ICSSBE), Langkawi, Malaysia.

[52] Bujang M.A., Ab-Ghani P., Soelar S.A., Zulkifli N.A., Omar E.D. (2019). Invalid skewed responses contribute to invalid factor solution in exploratory factor analysis: a validation approach using real-life data. J. Behav. Health..

[53] Coan R.W. (1959). A comparison of oblique and orthogonal factor solutions. J. Exp. Educ..

[54] Kline R.B. (2016).

[55] (2015). Merriam-webster dictionary. Encyclopædia Britannica Online.

[56] Constitution W.H.O. (2023). https://www.who.int/about/governance/constitution.

[57] (2015). Merriam-webster dictionary. Encyclopædia Britannica Online.

[58] Skevington S.M., Rowland C., Panagioti M., Bower P. (2021). Krägeloh C. Enhancing the multi-dimensional assessment of quality of life: introducing the WHOQOL-Combi. Qual. Life Res..

[59] (2015). Merriam-webster dictionary. Encyclopædia Britannica Online.

[60] (2015). Merriam-webster dictionary. Encyclopædia Britannica Online.

[61] Abraham L., Tara S., Mark F.M. (2008). Psychometric validation of a sexual quality of life questionnaire for use in men with premature ejaculation or erectile dysfunction. J. Sex. Med..

