# Supplementary material for: Measuring population health and quality of life: Developing and testing of the significant quality of life measure (SigQOLM)
Source: Heliyon. 2023 Nov 29;9(12):e22668. doi: 10.1016/j.heliyon.2023.e22668 (PMC10750041; doi:10.1016/j.heliyon.2023.e22668)
Supplement: Multimedia component 1 [file mmc1.docx]

**Significant Quality of Life Measures (SigQOLM) – Past TWO weeks**

***Pengukuran Kualiti Hidup yang Bermakna – DUA minggu yang lepas***

**Instruction:**

**The aim of this questionnaire is to measure how you feel about your quality of life-related to health and non-health conditions for the past TWO weeks. You will be asked how frequently certain things happened in your life for the past TWO weeks. Please read the statements carefully and mark (x) as the most appropriate option for you.**

***Arahan:***

***Soal selidik ini bertujuan untuk mengukur kualiti hidup seseorang daripada keadaan kesihatan dan bukan kesihatan untuk DUA minggu yang lepas. Anda akan ditanya setakat mana kekerapan sesuatu perkara itu berlaku dalam hidup anda dalam masa DUA minggu yang lepas. Sila baca setiap soalan dengan teliti dan tandakan (x) pilihan terbaik anda.***

| No. | Item / *Perkara* | Never  *Tidak pernah* | Seldom  *Jarang* | Sometimes  *Kadang-kadang* | Normally  *Biasanya* | Always  *Sentiasa* |
| --- | --- | --- | --- | --- | --- | --- |
|  | **SigQOLM – HEALTH** |  |  |  |  |  |
|  | 1. **Physical pain / *Kesakitan fizikal*** |  |  |  |  |  |
| 1 | I feel discomfort at any part of my body  *Saya rasa tidak selesa di mana-mana bahagian badan saya* | ( ) | ( ) | ( ) | ( ) | ( ) |
| 2 | I have to take medications  *Saya perlu makan ubat* | ( ) | ( ) | ( ) | ( ) | ( ) |
| 3 | I have to see a doctor  *Saya perlu berjumpa doktor* | ( ) | ( ) | ( ) | ( ) | ( ) |
| 4 | I feel pain at any part of my body  *Saya rasa sakit di mana-mana bahagian badan saya* | ( ) | ( ) | ( ) | ( ) | ( ) |
| 5 | I feel unhealthy  *Saya rasa tidak sihat* | ( ) | ( ) | ( ) | ( ) | ( ) |
|  | 1. **Physical energy/ *Tenaga fizikal*** |  |  |  |  |  |
| 6 | I am easily exhausted when I do my daily activities (e.g. walking, working, etc)  *Saya mudah keletihan apabila melakukan aktiviti harian (cth. berjalan, bekerja, dll)* | ( ) | ( ) | ( ) | ( ) | ( ) |
| 7 | I am unable to carry heavy items which I used to be able to do previously  *Saya tidak dapat memikul barang berat seperti yang saya biasa lakukan sebelum ini* | ( ) | ( ) | ( ) | ( ) | ( ) |
| 8 | I am unable to perform heavy task which I used to be able to do previously  *Saya tidak dapat melakukan kerja berat seperti yang biasa saya lakukan sebelum ini* | ( ) | ( ) | ( ) | ( ) | ( ) |
| 9 | I feel lack of physical energy  *Saya rasa kurang tenaga fizikal* | ( ) | ( ) | ( ) | ( ) | ( ) |
|  | **3.0 Emotional symptoms/ simptom *emosi*** |  |  |  |  |  |
| 10 | I feel depressed  *Saya berasa tertekan* | ( ) | ( ) | ( ) | ( ) | ( ) |
| 11 | I feel anxious  *Saya berasa cemas* | ( ) | ( ) | ( ) | ( ) | ( ) |
| 12 | I am not motivated to pursue any activities  *Saya tidak bersemangat untuk melakukan sebarang aktiviti* | ( ) | ( ) | ( ) | ( ) | ( ) |
|  | **4.0 Independent/ *Kemampuan untuk berdikari*** |  |  |  |  |  |
| 13 | I depend on others to move from one place to another  *Saya bergantung kepada orang lain untuk bergerak dari satu tempat ke satu tempat yang lain* | ( ) | ( ) | ( ) | ( ) | ( ) |
| 14 | I need assistance from others to help me in my daily activities  *Saya memerlukan bantuan daripada orang lain untuk membantu saya melakukan aktiviti-aktiviti harian* | ( ) | ( ) | ( ) | ( ) | ( ) |
| 15 | I am dependent on others to fulfil my self-care needs  *Saya bergantung kepada orang lain untuk memenuhi keperluan penjagaan diri saya* | ( ) | ( ) | ( ) | ( ) | ( ) |
|  | **5.0 Mobility / *Pergerakan*** |  |  |  |  |  |
| 16 | I need an equipment (eg: walking stick, wheelchair, etc) to help me mobilize  *Saya memerlukan sesuatu peralatan (cth: tongkat, kerusi roda, dll) untuk bergerak* | ( ) | ( ) | ( ) | ( ) | ( ) |
| 17 | My movements are slower than people of my age  *Pergerakan saya lebih lambat daripada orang yang seusia dengan saya* | ( ) | ( ) | ( ) | ( ) | ( ) |
| 18 | I have difficulty climbing stairs  *Saya menghadapi kesukaran untuk menaiki tangga* | ( ) | ( ) | ( ) | ( ) | ( ) |
| 19 | I feel pain when I move  *Saya rasa sakit apabila saya bergerak* | ( ) | ( ) | ( ) | ( ) | ( ) |
|  | **6.0 Sleep quality/ *Kualiti tidur*** |  |  |  |  |  |
| 20 | I am not satisfied with my sleep quality  *Saya tidak berpuas hati dengan kualiti tidur saya* | ( ) | ( ) | ( ) | ( ) | ( ) |
| 21 | I have difficulty falling asleep  *Saya mengalami kesukaran untuk tidur* | ( ) | ( ) | ( ) | ( ) | ( ) |
| 22 | I sleep less than 6 hours a night  *Saya tidur kurang dari 6 jam pada waktu malam* | ( ) | ( ) | ( ) | ( ) | ( ) |
| 23 | I feel tired after I wake up from sleep  *Saya berasa letih selepas bangun daripada tidur* | ( ) | ( ) | ( ) | ( ) | ( ) |
|  | **7.0 Eating regime/ *Aturan pemakanan*** |  |  |  |  |  |
| 24 | I am careful with what I eat  *Saya berhati-hati dengan apa yang saya makan* | ( ) | ( ) | ( ) | ( ) | ( ) |
| 25 | I need to control my diet  *Saya perlu mengawal diet (pemakanan) saya* | ( ) | ( ) | ( ) | ( ) | ( ) |
|  | **8.0 Body image / *Imej tubuh badan*** |  |  |  |  |  |
| 26 | I am not satisfied with my body image  *Saya tidak berpuas hati dengan imej bentuk badan saya* | ( ) | ( ) | ( ) | ( ) | ( ) |
| 27 | I am not satisfied with my weight  *Saya tidak berpuas hati dengan berat saya* | ( ) | ( ) | ( ) | ( ) | ( ) |
| 28 | I wish to change my body image  *Saya berharap dapat menukar bentuk imej badan saya* | ( ) | ( ) | ( ) | ( ) | ( ) |
| 29 | I don’t like my present body appearance  *Saya tidak suka penampilan saya sekarang* | ( ) | ( ) | ( ) | ( ) | ( ) |
|  | **9.0 Perception of future health / *Persepsi kesihatan di masa hadapan*** |  |  |  |  |  |
| 30 | I am worried that I will suffer poor health within 5 years  *Saya bimbang saya akan menghadapi kesihatan yang teruk dalam masa 5 tahun* | ( ) | ( ) | ( ) | ( ) | ( ) |
| 31 | I am worried that my lifespan is shorter than people of my age  *Saya bimbang jangka hayat kehidupan saya lebih pendek daripada orang seusia saya* | ( ) | ( ) | ( ) | ( ) | ( ) |
| 32 | I am worried that my health will not improve  *Saya bimbang kesihatan saya tidak akan bertambah baik* | ( ) | ( ) | ( ) | ( ) | ( ) |
| 33 | I have no solution to improve my health  *Saya tidak mempunyai penyelesaian untuk menambahbaik keadaan kesihatan saya* | ( ) | ( ) | ( ) | ( ) | ( ) |
|  | **SigQOLM – RELATIONSHIP** |  |  |  |  |  |
|  | **10.0 Family relationships/ *Hubungan dengan ahli keluarga*** |  |  |  |  |  |
| 1 | My family does not care about me  *Keluarga saya tidak mengambil berat tentang diri saya* | ( ) | ( ) | ( ) | ( ) | ( ) |
| 2 | I am not satisfied with my family relationships  *Saya tidak berpuas hati dengan hubungan kekeluargaan saya* | ( ) | ( ) | ( ) | ( ) | ( ) |
| 3 | My family does not provide aid when I need their help  *Keluarga saya tidak menghulurkan bantuan apabila saya memerlukan bantuan* | ( ) | ( ) | ( ) | ( ) | ( ) |
| 4 | I am not involved in my family activities  *Saya tidak terlibat dengan aktiviti-aktiviti kekeluargaan saya* | ( ) | ( ) | ( ) | ( ) | ( ) |
|  | **11.0 Friendship / *Persahabatan*** |  |  |  |  |  |
| 5 | I notice my friends are uncomfortable befriending me  *Saya mendapati kawan-kawan saya tidak selesa berkawan dengan saya* | ( ) | ( ) | ( ) | ( ) | ( ) |
| 6 | My friends ignore me  *Kawan-kawan saya tidak mempedulikan saya* | ( ) | ( ) | ( ) | ( ) | ( ) |
| 7 | My friends do not appreciate my friendship  *Kawan-kawan saya tidak menghargai persahabatan saya* | ( ) | ( ) | ( ) | ( ) | ( ) |
| 8 | My friends do not want to help me when I am in trouble  *Kawan-kawan saya tidak mahu membantu saya apabila saya dalam kesusahan* | ( ) | ( ) | ( ) | ( ) | ( ) |
|  | **12.0 Religiosity / *Keagamaan*** |  |  |  |  |  |
| 9 | I am not interested to study my religion  *Saya tidak berminat untuk belajar tentang agama saya* | ( ) | ( ) | ( ) | ( ) | ( ) |
| 10 | I do not practise my religion in my daily life  *Saya tidak mempraktikkan amalan agama dalam kehidupan seharian saya* | ( ) | ( ) | ( ) | ( ) | ( ) |
| 11 | I find peace not from religious practices  *Saya mendapat ketenangan bukan daripada amalan-amalan agama* | ( ) | ( ) | ( ) | ( ) | ( ) |
| 12 | I do what I want without referring to religious teachings  *Saya lakukan apa yang saya mahu tanpa merujuk kepada ajaran-ajaran agama* | ( ) | ( ) | ( ) | ( ) | ( ) |
|  | **SigQOLM – FUNCTIONAL ACTIVITIES** |  |  |  |  |  |
|  | **13.0 Self-care / *Mengurus diri*** |  |  |  |  |  |
| 1 | I have problem getting dressed  *Saya menghadapi masalah untuk memakai pakaian* | ( ) | ( ) | ( ) | ( ) | ( ) |
| 2 | I have problem attending to my self-care needs  *Saya menghadapi masalah untuk mengurus keperluan diri sendiri* | ( ) | ( ) | ( ) | ( ) | ( ) |
| 3 | I have problem preparing food for myself  *Saya menghadapi masalah untuk menyediakan makanan untuk diri sendiri* | ( ) | ( ) | ( ) | ( ) | ( ) |
| 4 | I have problem taking a bath  *Saya menghadapi masalah untuk mandi* | ( ) | ( ) | ( ) | ( ) | ( ) |
| 5 | I have problem doing household chores  *Saya menghadapi masalah untuk melakukan kerja-kerja rumah* | ( ) | ( ) | ( ) | ( ) | ( ) |
|  | **14.0 Social life/ *Kehidupan sosial*** |  |  |  |  |  |
| 6 | I prefer to be alone  *Saya lebih suka bersendirian* | ( ) | ( ) | ( ) | ( ) | ( ) |
| 7 | I find social activities uncomfortable  *Saya rasa tidak selesa dengan activiti-aktiviti sosial* | ( ) | ( ) | ( ) | ( ) | ( ) |
| 8 | I feel awkward when I am with others  *Saya rasa janggal apabila bersama dengan orang lain* | ( ) | ( ) | ( ) | ( ) | ( ) |
|  | **15.0 Perception on time usage/ *Persepsi terhadap penggunaan masa*** |  |  |  |  |  |
| 9 | I am not doing any activities that can benefit me  *Saya tidak melakukan apa-apa aktiviti yang boleh memberi manfaat kepada saya* | ( ) | ( ) | ( ) | ( ) | ( ) |
| 10 | I am wasting my time doing unimportant things  *Saya membazir masa melakukan perkara-perkara yang tidak penting* | ( ) | ( ) | ( ) | ( ) | ( ) |
| 11 | The way I spend my time will not benefit others  Cara *saya menghabiskan masa tidak akan mendatangkan manfaat kepada orang lain* | ( ) | ( ) | ( ) | ( ) | ( ) |
| 12 | I am not using my time effectively to improve my life  *Saya tidak menggunakan masa dengan berkesan untuk menambah baik kehidupan saya* | ( ) | ( ) | ( ) | ( ) | ( ) |
| 13 | I am not satisfied with the way I use my time  *Saya tidak berpuas hati dengan cara saya menggunakan masa saya* | ( ) | ( ) | ( ) | ( ) | ( ) |
|  | **SigQOLM – SURVIVAL** |  |  |  |  |  |
|  | **16.0 Basic needs / *Keperluan asas*** |  |  |  |  |  |
| 1 | I have to borrow money to fulfill my basic needs  *Saya perlu meminjam wang untuk memenuhi keperluan asas saya* | ( ) | ( ) | ( ) | ( ) | ( ) |
| 2 | I am not satisfied with my own basic needs  *Saya tidak berpuas hati dengan keperluan asas yang saya miliki* | ( ) | ( ) | ( ) | ( ) | ( ) |
| 3 | I am unable to fulfill my basic needs  *Saya tidak berupaya untuk memenuhi keperluan asas saya* | ( ) | ( ) | ( ) | ( ) | ( ) |
|  | **17.0 Safety / Keselamatan** |  |  |  |  |  |
| 4 | I live in an unsafe environment  *Saya menetap di satu persekitaran yang tidak selamat* | ( ) | ( ) | ( ) | ( ) | ( ) |
| 5 | I feel unsafe whenever I leave my house  *Saya rasa tidak selamat apabila keluar dari rumah* | ( ) | ( ) | ( ) | ( ) | ( ) |
| 6 | I feel that my safety can be threatened at any time  *Saya rasa keselamatan saya boleh terancam pada bila – bila masa* | ( ) | ( ) | ( ) | ( ) | ( ) |
| 7 | I fear for my safety  *Saya khuatir tentang keselamatan saya* | ( ) | ( ) | ( ) | ( ) | ( ) |
|  | **18.0 Perception of future conditions / *Persepsi kondisi di masa depan*** |  |  |  |  |  |
| 8 | I am worried that I will have a bad future  *Saya bimbang yang saya akan mempunyai masa depan yang teruk* | ( ) | ( ) | ( ) | ( ) | ( ) |
| 9 | It is already too late to improve my life’s condition  *Keadaan kehidupan saya sudah terlalu lambat untuk dipulihkan* | ( ) | ( ) | ( ) | ( ) | ( ) |
| 10 | I have lost hope in life  *Saya telah hilang harapan dalam kehidupan* | ( ) | ( ) | ( ) | ( ) | ( ) |
| 11 | I do not have solutions to improve my future  *Saya tidak mempunyai penyelesaian untuk membaik pulih masa depan saya* | ( ) | ( ) | ( ) | ( ) | ( ) |
